# Stroke in Sierra Leone: Case fatality rate and functional outcome after stroke in Freetown

**DOI:** 10.1177/17474930231164892

**Published:** 2023-03-25

**Authors:** Daniel Youkee, Gibrilla F Deen, Mamadu Baldeh, Zainab F Conteh, Julia Fox-Rushby, Musa Gbessay, Jotham Johnson, Peter Langhorne, Andrew JM Leather, Durodami R Lisk, Iain J Marshall, Jessica O’Hara, Sahr Pessima, Anthony Rudd, Marina Soley-Bori, Melvina Thompson, Hatem Wafa, Yanzhong Wang, Caroline L Watkins, Christine E Williams, Charles DA Wolfe, Catherine M Sackley

**Affiliations:** 1 School of Life Course & Population Sciences, King’s College London, London, UK; 2 College of Medicine and Allied Health Sciences, The University of Sierra Leone, Freetown, Sierra Leone; 3 Connaught Teaching Hospital, University of Sierra Leone Teaching Hospital Complex, Freetown, Freetown, Sierra Leone; 4 Collaboration for Leadership in Applied Health Research and Care South London, National Institute for Health Research, London, UK; 5 34 Military Hospital, Freetown, Sierra Leone; 6 Stroke Association Sierra Leone, Freetown, Sierra Leone; 7 Academic Section of Geriatric Medicine, School of Cardiovascular & Metabolic Health, University of Glasgow, Glasgow, UK; 8 King’s Centre for Global Health and Health Partnerships, School of Life Course & Population Sciences, King’s College London, London, UK; 9 Faculty of Health and Care, University of Central Lancashire, Preston, UK; 10 Faculty of Medicine and Health Sciences, University of Nottingham, Nottingham, UK

**Keywords:** Stroke, Sierra Leone, Africa, case fatality rate, function, outcomes, Barthel index

## Abstract

**Background:**

There is limited information on long-term outcomes after stroke in sub-Saharan Africa (SSA). Current estimates of case fatality rate (CFR) in SSA are based on small sample sizes with varying study design and report heterogeneous results.

**Aims:**

We report CFR and functional outcomes from a large, prospective, longitudinal cohort of stroke patients in Sierra Leone and describe factors associated with mortality and functional outcome.

**Methods:**

A prospective longitudinal stroke register was established at both adult tertiary government hospitals in Freetown, Sierra Leone. It recruited all patients ⩾ 18 years with stroke, using the World Health Organization definition, from May 2019 until October 2021. To reduce selection bias onto the register, all investigations were paid by the funder and outreach conducted to raise awareness of the study. Sociodemographic data, National Institute of Health Stroke Scale (NIHSS), and Barthel Index (BI) were collected on all patients on admission, at 7 days, 90 days, 1 year, and 2 years post stroke. Cox proportional hazards models were constructed to identify factors associated with all-cause mortality. A binomial logistic regression model reports odds ratio (OR) for functional independence at 1 year.

**Results:**

A total of 986 patients with stroke were included, of which 857 (87%) received neuroimaging. Follow-up rate was 82% at 1 year, missing item data were <1% for most variables. Stroke cases were equally split by sex and mean age was 58.9 (SD: 14.0) years. About 625 (63%) were ischemic, 206 (21%) primary intracerebral hemorrhage, 25 (3%) subarachnoid hemorrhage, and 130 (13%) were of undetermined stroke type. Median NIHSS was 16 (9–24). CFR at 30 days, 90 days, 1 year, and 2 years was 37%, 44%, 49%, and 53%, respectively. Factors associated with increased fatality at any timepoint were male sex (hazard ratio (HR): 1.28 (1.05–1.56)), previous stroke (HR: 1.34 (1.04–1.71)), atrial fibrillation (HR: 1.58(1.06–2.34)), subarachnoid hemorrhage (HR: 2.31 (1.40–3.81)), undetermined stroke type (HR: 3.18 (2.44–4.14)), and in-hospital complications (HR: 1.65 (1.36–1.98)). About 93% of patients were completely independent prior to their stroke, declining to 19% at 1 year after stroke. Functional improvement was most likely to occur between 7 and 90 days post stroke with 35% patients improving, and 13% improving between 90 days to 1 year. Increasing age (OR: 0.97 (0.95–0.99)), previous stroke (OR: 0.50 (0.26–0.98)), NIHSS (OR: 0.89 (0.86–0.91)), undetermined stroke type (OR: 0.18 (0.05–0.62)), and ⩾1 in-hospital complication (OR: 0.52 (0.34–0.80)) were associated with lower OR of functional independence at 1 year. Hypertension (OR: 1.98 (1.14–3.44)) and being the primary breadwinner of the household (OR: 1.59 (1.01–2.49)) were associated with functional independence at 1 year.

**Conclusion:**

Stroke affected younger people and resulted in high rates of fatality and functional impairment relative to global averages. Key clinical priorities for reducing fatality include preventing stroke-related complications through evidence-based stroke care, improved detection and management of atrial fibrillation, and increasing coverage of secondary prevention. Further research into care pathways and interventions to encourage care seeking for less severe strokes should be prioritized, including reducing the cost barrier for stroke investigations and care.

## Introduction

It is estimated that stroke is the second leading cause of adult death in sub-Saharan Africa (SSA), and a cause of significant morbidity.^1^ However, prospective stroke studies of case fatality rate and functional outcome in SSA are limited in number and quality,^2^ and long-term outcomes after stroke have not been previously studied in Sierra Leone. Understanding survival and functional outcome after stroke is important to provide prognostic information for the patient, characterize the natural history of stroke and to inform health system planning to meet the acute and long-term care needs of patients after stroke.^3^

The evidence base for case fatality rate (CFR) after stroke in SSA is heterogeneous and of varying quality.^4^ Available data suggest that mortality is higher in some African countries than global estimates.^5^ A systematic review of CFR in SSA, published in 2021, included 91 studies^6^ with 9 (9.9%) studies rated as high-quality evidence. CFR at 90 days was 22.3% (95% confidence interval (CI): 16.6–29.2) and at 1 year was 33.2% (95% CI: 23.6–44.5). For 1 year CFR, the review included a pooled sample size of 4809 patients with CFR displaying high heterogeneity ranging from 9.5% to 75.4% across studies. In contrast, a recent prospective hospital study of 564 stroke patients in Ghana found CFR at 3 months and 1 year of 43.2% and 49.7%, respectively.^7^ This compares to global 30-day CFR of 17–30% reported in high-income countries and 18–35% in low- and middle-income countries.^8^ Worldwide, CFR differs by stroke type, generally being higher for intracerebral hemorrhage compared with ischemic stroke at 1 year.^3,9^ Evidence suggests that prevalence of stroke type and stroke subtypes prevalence differ in SSA, with higher proportions of intracerebral hemorrhage, higher proportions of small vessel disease, and lower proportions of cardioembolic ischemic stroke subtypes, compared with other regions.^5,10,11^ The different prevalence of stroke types and subtypes may impact case fatality, indeed pooled estimates of CFR at 1year in SSA are higher for ischemic stroke compared with intracerebral hemorrhage.^6^ Older age is a nonmodifiable risk factor for stroke strongly associated with both increased case fatality and worse functional outcome after stroke in other regions.^12^ However, stroke occurs at a younger age in SSA^13^ compared with other regions and life expectancy at birth also differs.^5^ Previous work in Sierra Leone suggests stroke occurs at median age of 59 years,^14^ in the context of an adult life expectancy at birth of 60 years, 20 years younger than other regions.^15^

Due to differences in demographics, prevalence of stroke types, and access to high-quality stroke care, we hypothesize that CFR and variables associated with CFR may differ in Sierra Leone compared with global rates and associations. A previous retrospective hospital-based study in Sierra Leone found in-hospital mortality to be associated with prior stroke, hypertension, hemorrhagic stroke, and aspiration pneumonia,^16^ while a prospective study at the same hospital found male sex, pneumonia, subarachnoid hemorrhage, and undetermined stroke types were associated with in-hospital death.^14^ In this article, we describe long-term case fatality and function after stroke in Sierra Leone and identify factors associated with survival and functional outcome.

## Methods

A prospective stroke register was established at the two adult tertiary government hospitals in Freetown, Sierra Leone: at Connaught Teaching Hospital from 1 May 2019 until 30 September 2021 and at 34 Military Hospital from 1 February 2021 until 2 September 2021. All patients 18 years and over meeting the World Health Organization ICD10 definition of stroke were included. The study methods and the health care setting have been previously described.^14^ All stroke subtypes were included: ischemic (ICD63); intracerebral hemorrhage (ICD61); subarachnoid hemorrhages (ICD60); and undetermined stroke types (ICD62).^17^ Classification of pathological stroke subtype, using the Oxford Community Stroke Project (OCSP) classification,^18^ was conducted by an experienced stroke physician, with reference to the case history, investigation results, and imaging. During the study period, stroke investigations, including CT scanning, were funded by the grant, National Institute for Health Research (NIHR) (GHR:17:63:66), and provided free of charge to patients, to reduce bias in access to investigations. During the study period, there was no functional CT scanner at either of the hospitals, so CT scanning was provided at two off-site private radiology centers. The study supported ambulance transfer accompanied by a clinician to enable safe access to imaging. Participants who did not receive neuroimaging (often in practice due to being too critically ill to transfer for scanning) or those in whom neuroimaging was not conclusive were classified as undetermined stroke type.

Participants were followed up at 90 days, 1 year and 2 years post stroke. Patients were primarily contacted by telephone, and those uncontactable were visited at home. We report on follow-up from 1 May 2019 to 22 July 2022. All-cause mortality was recorded from hospital records and as reported by caregiver or relative at follow-up. Functional outcome was measured using the Barthel Index (BI) and was retrospectively reported by patients and family 7 days prior to stroke, then measured at 7 days post stroke, 90 days post stroke, at 1 year, and each year after stroke. BI was categorized as completely dependent (BI < 60), dependent (BI = 60–84), independent with assistance BI ⩾ 85,^19^ and independent without assistance as BI = 100.

Survival curves were constructed for the whole population and for subgroups by stroke subtype, age, and sex; all using the Kaplan–Meier method. Multivariable Cox proportional hazards models were conducted to assess the independent effect of variables on all-cause mortality. A logistic regression model reporting odds ratios (OR) was created for functional independence at 1 year. A full description of stroke type classification, risk factors, missing data, and regression model development is provided in the appendix.

All data were collected on standardized paper case report forms. Double data entry was conducted, and all data uploaded onto REDCap™.^20^ Statistical analyses were performed in STATA v17, StataCorp™.^21^ The study received ethical approval from King’s College London (HR-18/19-8467) and approval from the Sierra Leone Ethical and Scientific Review Committee on 18 December 2018. Written consent was sought from all patients. For those judged not to have capacity, informed consent was sought from the next of kin.

## Results

The register recruited 1145 people with suspected strokes. After clinical review and neuroimaging, 986 were confirmed as strokes and were maintained as the core population for analysis, 915 at Connaught Teaching Hospital and 71 at 34 Military Hospital. Stroke cases were equally split by sex, and mean age was 58.9 (SD: 14.0) years. During the study period, there was no stroke unit at either hospital and no patients received thrombolytic therapy or mechanical thrombectomy. The median time from stroke onset to admission was 24 hours and median length of stay was 7 days (interquartile range: 3–12). A total of 857 (87%) patients underwent neuroimaging: 847 received CT scans and 10 received MRI; comparative statistics of patients who received neuroimaging versus those who did not are presented in the supplementary material. Median National Institute of Health Stroke Scale (NIHSS) was 16 (9–24). A total of 625 (63%) patient had ischemic stroke, 206 (21%) primary intracerebral hemorrhage, 25 (3%) subarachnoid hemorrhage, and 130 (13%) were of undetermined stroke type. Ischemic stroke subtypes by OCSP classification are described in Supplementary Figure One. A total of 355 (36.0%) patients died in hospital during the initial admission and 175 (17.7%) post hospital discharge. At 1 year, 182 (18.5%) participants were lost to follow-up; follow-up counts are reported in Supplementary Table One. Missing item data are reported in Supplementary Table Two, and were low at less than 1% for most variables.

### Case fatality rate

Case fatality rate was 37.1% at 30 days, 44.4% at 90 days, 49.9% at 1 year, and 53.2% at 2 years (Table 1). Case fatality rates by stroke type are shown in Table 1 and univariable analysis of CFR at 1 year is reported in Supplementary Table Three. Ischemic stroke CFR increased from 25.3% at 30 days to 45.6% at 2 years, while intracerebral hemorrhage CFR increased from 40.3% at 30 days to 51.0% at 2 years.

**Table 1.** Case fatality rate by stroke type, at 30 days, 90 days, 1 year, and 2 years post stroke.

Case fatality	All strokes	Ischemic	Intracerebral hemorrhage	Subarachnoid hemorrhage	Undetermined
	N = 986	N = 625 (63%)	N = 206 (21%)	N = 25 (3%)	N = 130 (13%)
30-day case fatality	366 (37.1%)	158 (25.3%)	83 (40.3%)	15 (60%)	110 (84.6%)
90-day case fatality	438 (44.4%)	210 (33.6%)	91 (44.2%)	18 (72%)	119 (91.5%)
One-year case fatality	492 (49.9%)	258 (41.3%)	95 (46.1%)	18 (72%)	121 (93.1%)
Two-year case fatality	529 (53.2%)	285 (45.6%)	105 (51.0%)	18 (72%)	121 (93.1%)

**Figure 1.**
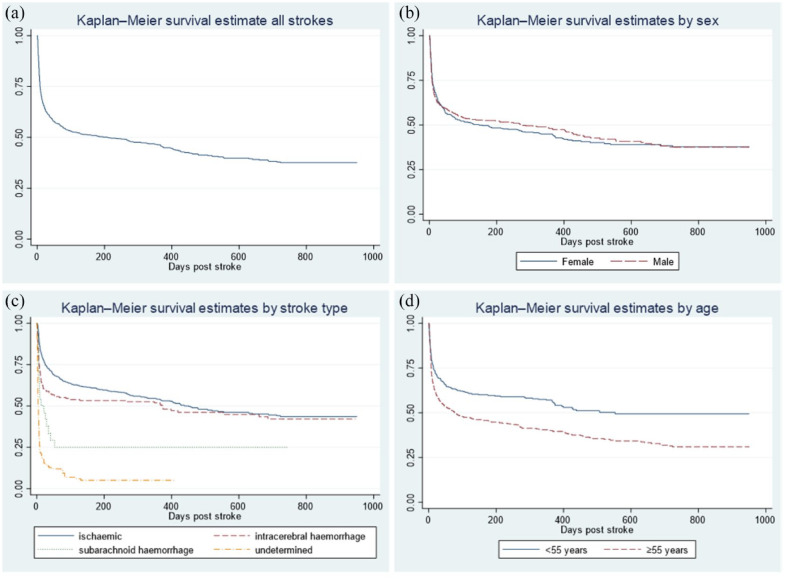
Kaplan–Meier estimates for stroke survival from date of stroke onset. (a) Kaplan–Meier survival estimate for all strokes (n = 986). (b) Kaplan–Meier survival estimate by sex (n = 986); logrank test p = 0.75. (c) Kaplan–Meier survival estimate by stroke type (n = 986); logrank test p = 0.0001. (d) Kaplan–Meier survival estimate by age < 55 years versus ⩾ 55 years (n = 981); logrank test p = 0.0001.

Kaplan–Meier survival estimates are shown in Figure 1. The survival estimates demonstrate that most deaths occur within the first 90 days post stroke; however, survival continues to decrease up to 2 years post stroke. Figure 1(c) demonstrates significant differences in survival by stroke type (p = 0.0001), with intracerebral hemorrhage patients initially having a steep decrease in survival compared with ischemic stroke patients, followed by survival estimates for the two stroke types becoming closer over time. Figure 1(d) demonstrates significantly reduced survival in patients ⩾ 55 years (p = 0.0001). Kaplan–Meier survival curves with censoring hashmarks are reported in Supplementary Figure Two. Univariable analysis of death at 1 year by stroke type is reported in Supplementary Table Four.

Cox proportional hazards model for fatality is shown in Table 2. Male sex, previous stroke, atrial fibrillation, subarachnoid hemorrhage, undetermined stroke type, and hospital complication were all associated with death. The presence of hypertension was associated with survival. A sensitivity analysis excluding in-hospital complications is presented in Supplementary Table Six.

**Table 2.** Cox proportional hazards model for fatality for all strokes.

Independent variable	Count	Hazard ratio	95% CI
Age (each additional year)^a^	58.9 (SD: 14.0)	1.00	1.00–1.01
Male sex	495 (50.2%)	1.28	1.05–1.56
Previous stroke	128 (13.0%)	1.34	1.04–1.71
Hypertension	831 (84.3%)	0.71	0.57–0.90
Diabetes	212 (21.5%)	1.04	0.84–1.29
Atrial fibrillation	38 (3.9%)	1.58	1.06–2.34
Dyslipidemia	401 (40.7%)	—	
Current smoker	153 (15.8%)	—	
Alcohol use (any)	255 (26.7%)	—	
Resident of Freetown	822 (84.0%)	—	
Higher education level	367 (37.2%)	0.91	0.75–1.10
Primary breadwinner	424 (43%)	0.88	0.72–1.07
Pre-stroke Barthel Index Mean (SD)	96.7 (12.4)	—	
NIHSS (each additional point)^a^	16 (IQR: 9-24)	1.07	1.06–1.08
Intracerebral hemorrhage^b^	206 (20.9%)	1.18	0.93–1.50
Subarachnoid hemorrhage^b^	25 (2.5%)	2.31	1.40–3.81
Undetermined stroke type^b^	130 (13.2%)	3.18	2.44–4.14
⩾ 1 in-hospital complication	396 (40.2%)	1.65	1.36–1.98

Variables with dashes (—) were not included in regression model.

CI: confidence interval; NIHSS: National Institute of Health Stroke Scale; IQR: interquartile range.

a Control variables.

b Stroke type compared with ischemic stroke n = 986.

### Functional outcome

**Figure 2.**
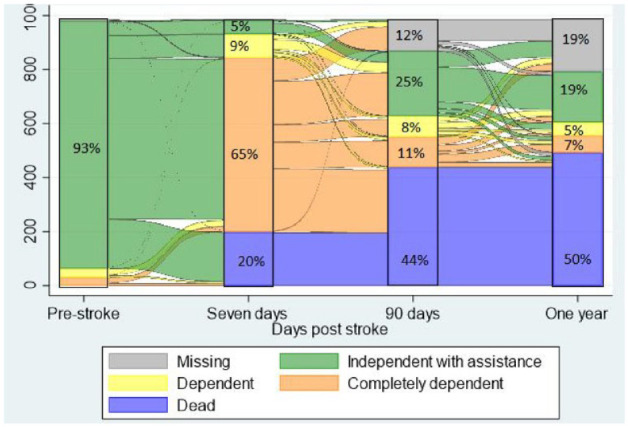
Sankey plot of flow of functional outcome pre-stroke and 7 days, 90 days, and 1 year post stroke. Percentage demonstrates proportion of patients with functional outcome measured by BI: Independent with assistance BI ⩾ 85, Dependent BI 60–84, Completely dependent BI < 60.

Progression of functional status displayed by Barthel Index (BI) at 7 days prior to stroke and 7 days, 90 days, and 1 year post stroke is shown in Figure 2. Seven days prior to stroke, 93% of patients were independent with assistance, 5% at 7 days post stroke, 28% at 90 days, and 19% at 1 year. From 7 days to 90 days, categorical functional status improved for 272 (34.8%) patients, worsened (including death) for 251 (32.1%), stayed the same for 145 (18.5%), and 114 (14.6%) were missing. From 90 days to 1 year, categorical functional status improved for 56 (13.0%), worsened (including death) for 92 (21.4%), stayed the same for 169 (39.3%), and 113 (26.3%) were missing.

Logistic regression with odds ratios (OR) for functional independence with assistance (BI ⩾ 85) at 1 year is shown in Table 3. Increasing age, previous stroke, NIHSS, undetermined stroke type and ⩾ 1 in-hospital complication were associated with lower OR of functional independence at 1 year, while hypertension and being the primary breadwinner of the household were associated with higher OR of functional independence at 1 year.

**Table 3.** Logistic regression for functional independence with assistance (BI ⩾ 85) at 1 year.

Independent variable	OR	95% CI
Age (each additional year)	0.97	0.95–0.99
Male sex	0.89	0.57–1.39
Previous stroke	0.50	0.26–0.98
Hypertension	1.98	1.14–3.44
Diabetes	0.64	0.39–1.05
Atrial fibrillation	0.73	0.20–2.66
Higher education level	1.32	0.86–2.01
Primary breadwinner	1.59	1.01–2.49
NIHSS (each additional point)	0.89	0.86–0.91
Intracerebral hemorrhage^a^	1.26	0.77–2.07
Subarachnoid hemorrhage^a^	0.36	0.11–1.23
Undetermined stroke type^a^	0.18	0.05–0.62
⩾ 1 in-hospital complication	0.52	0.34–0.80

BI: Barthel Index; OR: odds ratio; CI: confidence interval; NIHSS: National Institute of Health Stroke Scale.

a Stroke type compared with ischemic stroke.

## Discussion

This is the first publication reporting long-term outcomes after stroke in Sierra Leone. We report 30-day CFR of 37.1%, at the higher end of the global estimates of CFR.^22^ Ninety-day CFR of 44.4% and 1-year CFR of 49.7% are higher than pooled CFR estimates in SSA at 90 days of 22.3% (95% CI: 16.6–29.2) and 1 year of 33.2% (95% CI: 23.6–44.5), respectively.^6^ Our reported CFR is higher than similar studies in Benin,^23^ Kenya,^24^ and closely matches reported CFR in Ghana.^7^ The high CFR reported reflects the severe case mix; our median NIHSS was 16 (9–24), higher than other West African hospital-based stroke registers and significantly higher than seen in United Kingdom or North American hospital studies.^25^

Case fatality in our cohort was higher in patients with intracerebral hemorrhage strokes than ischemic strokes at all timepoints; the majority of deaths for patients with intracerebral hemorrhage occurred early in hospital, while ischemic stroke mortality was more evenly distributed across the study period. Relative to global pooled estimates by stroke type, ischemic stroke CFR was higher than expected and intracerebral hemorrhage CFR was within expected ranges. Our ischemic stroke 30-day CFR of 25% was higher than worldwide pooled estimates of 13.5% (95% CI 12.3–14.7) at 30 days.^26^ ICH mortality was similar to global estimates, 40% at 30 days compared with 36.3% (95% CI 31.5–41.2) and 46% at 1 year compared with 50.7% (95% CI = 45.2–56.2).^27^ This may partially reflect the under-detection of less severe ischemic strokes in our cohort, which may happen if people with less severe strokes choose not to seek care, due to common barriers in Sierra Leone such as cost of care,^28^ distance,^29^ trust in the formal health system^30^ and health literacy.^31^ Patients with intracerebral hemorrhage in our cohort were younger (Supplementary Table Four), had lower prevalence of diabetes, dyslipidemia, and atrial fibrillation, and higher pre-morbid status (pre-stroke BI) compared with ischemic strokes. Concurrently, it may reflect a lack of hyperacute stroke care for ischemic strokes and lack of access to quality care for the comorbidities more prevalent in ischemic stroke patients. Undetermined stroke types were associated with increased CFR as these patients were too sick to transfer for neuroimaging or died before neuroimaging could confirm stroke type (Supplementary Table Five). Subarachnoid hemorrhage CFR of 60% corresponds poorly to global estimates of CFR ranging from 27% to 44%,^32^ reported CFR of 40% in Sudan,^33^ 44.4% in Nigeria,^34^ and 45.6% in Kenya^35^ and reflects both the severity of strokes included in our cohort and lack of timely access to care, including neurosurgical intervention available in our setting.^36,37^

We report strokes occurring in younger people, with a mean age of 59 years. As in other settings, age was significantly associated with increased mortality, and patients alive at 1 year were on average 5 years younger than those dead at 1 year. Patients with a previous stroke had increased mortality (hazard ratio (HR): 1.34 (1.04–1.71), similar to other findings from SSA^6^ suggesting a need to improve the coverage and effectiveness of secondary prevention interventions in Sierra Leone. Atrial fibrillation was associated with increased mortality (HR: 1.58 (1.06–2.34)). However, only a single 12-lead electrocardiogram (ECG) was conducted in our cohort; therefore, atrial fibrillation was likely under-detected. Atrial fibrillation detection should be increased through use of cardiac holters or wearable smart devices with proven diagnostic accuracy.^38^ Atrial fibrillation diagnosis and management remains challenging in our setting, with the prohibitive costs of direct oral anticoagulants,^39^ and low levels of access to affordable and reliable International Normalized Ratio (INR) monitoring to allow safe and effective warfarin prescription.^40,41^ In-hospital complications were associated with fatality and worse functional outcome, demonstrating the need to implement evidence-based stroke care, such as stroke unit–based care, inclusive of swallow screening for the prevention of aspiration pneumonia.^14^

Our previous study found male sex to be associated with in-hospital fatality^14^; we demonstrate this finding again for long-term fatality (HR: 1.28 (1.05–1.56)). Further research is needed to understand whether this is due to intrinsic sex survival differences or potentially related to quality of care differences in the gender separated hospital wards. The presence of hypertension was associated with increased survival (HR: 0.71 (0.57–0.90)) in our cohort; pooled findings from SSA found no association between hypertension and CFR,^6^ while other West African prospective registers found a similar but non-significant direction of effect. This may demonstrate the higher relative fatality of stroke caused by other aetiologies, including renal disease, and malignancy, which were too small a sample size to include in our regression model. Alternatively, it may be due to the influence of hypertension on prevalence of subtypes of hemorrhagic stroke which may influence survival,^42 [Bibr bibr43-17474930231164892]^ which were not included in our regression model. Hypertension remains the primary dominant modifiable risk factor for stroke in our region,^10^ and hypertension detection, management, and control should be an urgent priority.^43^

Functional impairment was considerable; 93% of patients were completely independent 7 days prior to their stroke, and at 1 year post stroke, only 19% were independent with assistance. Most functional recovery was seen between 7 and 90 days, with 34.8% of patients reporting functional improvement, and a smaller proportion 13.0% improving from 90 days to 1 year. The functional improvement in some was matched by a greater amount of decrease in functional outcome and death in others, 32.1% worsening from 7 days to 90 days, and 21.4% from 90 days to 1 year. Rates of functional improvement appear similar to other studies in West Africa.^44,45^ Functional independence at 1 year was more likely in younger patients,^46^ first-in-a-lifetime strokes and being the primary breadwinner for the household. Socioeconomic status proxies, being the primary breadwinner (significantly) and higher educational attainment (non-significant), were associated with both improved survival and functional outcome at 1 year. This corresponds to regional^47^ and international findings^48^ and should inform the development of equitable stroke services in Sierra Leone.

### Strengths

This is the first study to report long-term CFR and functional status after stroke in Sierra Leone. The study benefits from a prospective, multi-center design and is one of the largest longitudinal studies of stroke patients in SSA to be published. Key elements of the stroke register were prospective methodology, community awareness raising and removal of the cost barrier for stroke investigations to reduce selection bias and increase access to care. During the study period, we recorded 381 strokes per year at Connaught Hospital, compared with 178 strokes per year in 2018.^16^ This is likely to include not only increased presentation of patients to the hospital but also increased awareness and recognition of stroke among health care workers and the removal of the cost barrier for investigations. Our study benefited from neuroimaging rates of 87% and a follow-up rate of 82% at 1 year.

### Limitations

The study is not population based; therefore, results cannot be extrapolated to the population level and are influenced by access to care. Selection bias onto the register, with care seeking only for severe strokes and under-detection of less severe strokes, likely contributes to the high CFR reported. Due to the lack of an onsite CT scanner, we were unable to provide imaging to the most critically ill patients with the most severe strokes (Supplementary Table Five); we therefore do not know the stroke subtype of the sickest patients in our cohort. The study is limited by not using an intracerebral hemorrhage classification system,^49^ such as the structural vascular lesions (S), medication (M), amyloid angiopathy (A), systemic disease (S), hypertension (H), or undetermined (U) (SMASH-U) criteria. Atrial fibrillation was only assessed by a single 12-lead ECG, therefore was likely under-detected.

## Conclusions

We demonstrate high CFR of 49.7% at 1 year relative to estimates in SSA and Europe. We report significant functional impairment; 93% of patients were completely independent 7 days prior to their stroke, and by 1 year post stroke, only 19% were independent with assistance. Key priorities include prevention of stroke-related complications through evidence-based interventions, such as stroke unit–based care, increased detection and improved management of atrial fibrillation, and enhanced coverage of secondary prevention, are key to reducing CFR after stroke in Sierra Leone. Further research into care pathways and interventions to encourage care seeking for less severe strokes should be prioritized.

## Supplemental Material

sj-docx-1-wso-10.1177_17474930231164892 – Supplemental material for Stroke in Sierra Leone: Case fatality rate and functional outcome after stroke in Freetown Click here for additional data file. Supplemental material, sj-docx-1-wso-10.1177_17474930231164892 for Stroke in Sierra Leone: Case fatality rate and functional outcome after stroke in Freetown by Daniel Youkee, Gibrilla F Deen, Mamadu Baldeh, Zainab F Conteh, Julia Fox-Rushby, Musa Gbessay, Jotham Johnson, Peter Langhorne, Andrew JM Leather, Durodami R Lisk, Iain J Marshall, Jessica O’Hara, Sahr Pessima, Anthony Rudd, Marina Soley-Bori, Melvina Thompson, Hatem Wafa, Yanzhong Wang, Caroline L Watkins, Christine E Williams, Charles DA Wolfe and Catherine M Sackley in International Journal of Stroke
