# Supplementary material for: Stroke in Sierra Leone: Case fatality rate and functional outcome after stroke in Freetown
Source: Int J Stroke. 2023 Mar 25;18(6):672–80. doi: 10.1177/17474930231164892 (PMC10311939; doi:10.1177/17474930231164892)
Supplement: sj-docx-1-wso-10.1177_17474930231164892 – Supplemental material for Stroke in Sierra Leone: Case fatality rate and functional outcome after stroke in Freetown [file sj-docx-1-wso-10.1177_17474930231164892.docx]

**Appendix One**

**Methods**

Due to limited access to primary healthcare, for many participants their stroke admission is their first encounter with the formal health care system and they present with undiagnosed risk factors. Therefore, we used risk factor definitions in line with previous international and regional stroke studies^1, 2^. Hypertension was defined as: blood pressure ≥140/90mmHg from 72 hours after stroke or; patient reported history of hypertension or; history of antihypertensive use or continuing use of antihypertensives 72 hours post stroke. Participants were categorised by their highest level of educational achievement. After sensitivity analysis, educational attainment was dichotomized as completion of high school, West African secondary school certificate examination (WASSCE), to allow comparison with other regional studies. Primary breadwinner was defined as the primary income earner in the household.

Clinician review of notes at discharge categorised patients as suffering a stroke-related complication or not. Complications assessed included pneumonia, urinary tract infection, seizures, deep vein thrombosis and pressure sores.

**Statistical analysis**

Missing data was assessed at the variable and participant level and at different follow-up timepoints. Multiple imputation was conducted to replace missing values. Continuous variables are reported as mean (SD) if normally distributed or as median (IQR) if non-normal distribution. Pearson’s Chi squared tests were used to examine associations between normal categorical variables, unpaired t-tests for continuous variables and Mann Whitney U test for non-normal distributions.

To inform model development, we conducted a literature review of variables associated with survival after stroke in SSA, see appendix one. Variable selection was informed by clinical gestalt and previous stroke studies in Sierra Leone^3, 4^ and SSA^5^. Two control variables were selected, NIHSS which has been associated with mortality after stroke in multiple settings^5-8^ and age. Full description of variable selection and model development is described in the appendix. Variables selected included sex, comorbidities, hypertension and diabetes, higher educational achievement, stroke type, and ≥1 in hospital complication. In hospital complications were included in the model, due to previous reporting of high prevalence of complications in this cohort, and on the assumption that a proportion of these complications are preventable. As complications may also be highly correlated with mortality, we conducted a sensitivity analysis excluding complications from the model (appendix one). Associations among variables were assessed via scatterplots and covariance matrices and assessed for multicollinearity by assessing variable inflation factors (appendix). As model diagnostics demonstrated that age violated the cox proportional hazards assumption, it was included as a time varying covariate.

*Supplementary Figure one: Flowchart of stroke types and Oxford Community Stroke Project (OCSP) classification. TACI, total anterior circulation infarction; PACI, partial anterior, circulation infarction; POCI, posterior circulation infarction; LACI, lacunar infarction; unclassified type.*

**Follow up counts**

|  | Contacted and alive | Verified alive at later timepoint | Total Alive | Total Dead | Lost to follow up |
| --- | --- | --- | --- | --- | --- |
| At discharge | 631 | - | 631 | 355 | 0 |
| 90 days | 433 | 18 | 451 | 438 | 115 (11.7%) |
| One year | 308 | 4 | 312 | 492 | 182 (18.5%) |

*Supplementary Table one: Follow up counts as of 3^rd^ October 2022*

**Missing Item data**

| Variable | Total | Missing | % Missing |
| --- | --- | --- | --- |
| Age | 981 | 5 | 0.51% |
| Sex | 986 | 0 | 0 |
| Educational level | 986 | 24 | 2.43% |
| NIHSS | 986 | 4 | 0.41% |
| District | 986 | 8 | 0.81% |
| Previous stroke | 986 | 5 | 0.51% |
| Hypertension | 986 | 0 | 0% |
| Diabetes | 986 | 1 | 0.1% |
| Atrial fibrillation | 986 | 0 | 0% |
| Dyslipidemia | 986 | 1 | 0.10% |
| Smoking | 986 | 15 | 1.52% |
| Alcohol | 986 | 31 | 3.14% |
| Previous medications prescribed | 986 | 29 | 2.94% |
| Barthel index pre stroke | 986 | 6 | 0.61% |
| Complications | 986 | 0 | 0% |
| Stroke type | 986 | 0 | 0% |

*Supplementary table two: Missing item data*

|  | **Died within one year (n=492)** | **Alive at one year (n=312)** | **P value** |
| --- | --- | --- | --- |
| **First in lifetime stroke** | **406 (82.7)** | **280 (90.6)** | **0.01** |
| Male | 238 (48.4) | 151 (48.4) | 0.06 |
| **Age mean (SD)** | **61.2 (13.9)** | **55.6 (13.1)** | **0.0001** |
| Resident of Freetown | 422 (85.8) | 263 (84.3) | 0.32 |
| Hypertension | 401 (81.5) | 271 (86.9) | 0.12 |
| Diabetes | 122 (24.8) | 61 (19.6) | 0.11 |
| Dyslipidaemia | 188 (38.2) | 133 (42.6) | 0.41 |
| Atrial Fibrillation | 28 (5.7) | 7 (2.2) | **0.05** |
| Current smoker | 71 (14.4) | 54 (17.3) | 0.57 |
| Alcohol use (any) | 126 (25.6) | 86 (27.6) | 0.58 |
| Increased waist hip ratio | 310 (63.0) | 209 (70.0) | 0.32 |
| Higher education level (finished high school) | 170 (34.6) | 128 (41.0) | 0.06 |
| **Primary breadwinner** | **185 (37.6)** | **150 (48.1)** | **0.003** |
| Prescribed Regular medicine before stroke (any) | 268 (54.4) | 163 (52.2) | 0.46 |
| **Pre-stroke Barthel Index Mean and (SD)** | **95.2 (14.4)** | **97.8 (10.7)** | **0.003** |
| **Mean National Institute of Health Stroke Severity Scale (SD)** | **21.3 (8.7)** | **11.5 (6.9)** | **0.0001** |
| Stroke type: Ischaemic  Intracerebral haemorrhage  Subarachnoid  Undetermined | 258 (52.4) | 231 (74.0) | **0.0001** |
|  | 95 (19.3) | 70 (22.4) |  |
|  | 18 (3.7) | 6 (1.9) |  |
|  | 121 (24.6) | 5 (1.6) |  |
| Mean BI (SD)seven days post stroke | 13.9 (21.0) | 40.5 (28.0) | **0.0001** |
| ≥1 In Hospital Complication  Pneumonia  Seizures  UTI  Pressure sores | 277 (56.3) | 80 (25.6) | **0.0001** |
|  | 179 (36.4) | 25 (8.0) | **0.0001** |
|  | 51 (10.4) | 13 (4.2) | **0.002** |
|  | 26 (5.3) | 19 (6.1) | 0.63 |
|  | 26 (5.3)) | 3 (1.0) | **0.001** |

*Supplementary table three: Univariate analysis by case fatality at one year, independent t tests, chi2 and Mann Whitney U test. Data is count (%), unless otherwise specified.*

|  | Ischaemic (n=625) | Intracerebral haemorrhage (n=206) | P value |
| --- | --- | --- | --- |
| First in lifetime stroke | 537 (85.9) | 183 (88.8) | 0.385 |
| Age mean (SD) | 60.9 (14.0) | 53.1 (12.0) | **0.0001** |
| Primary breadwinner | 262 (41.9) | 106 (51.5) | **0.024** |
| Hypertension | 516 (82.6) | 181 (87.9) | 0.073 |
| Diabetes | 151 (24.2) | 21 (10.2) | **0.0001** |
| Dyslipidaemia | 248 (39.7) | 93 (45.1) | **0.02** |
| Atrial Fibrillation | 30 (4.8) | 3 (1.5) | **0.033** |
| Current smoker  Ex smoker  Never smoked | 106 (17.0) | 27 (13.1) | 0.454 |
|  | 88 (14.1) | 31 (15.0) |  |
|  | 424 (67.8) | 143 (69.4) |  |
| Alcohol use (any) | 179 (28.6) | 46 (22.3) | 0.088 |
| Increased waist hip ratio | 428 (68.5) | 129 (62.6) | 0.182 |
| Higher education level (finished high school) | 247 (39.5) | 72 (35.0) | 0.276 |
| Prescribed Regular medicine before stroke (any) | 353 (56.5) | 78 (37.9) | **0.0001** |
| Pre-stroke Barthel Index Median and IQR | 100 (100-100) | 100 (100-100) | **0.0001** |
| National Institute of Health Stroke Severity Scale (median and IQR) | 13 (7-21) | 17 (11-25) | **0.0001** |
| Post stroke BI | 30 (5-50) | 15 (0-37.5) | **0.0001** |
| ≥1 In Hospital Complication  Pneumonia  UTI  Seizures  Pressure sores | 209 (33.4) | 81 (39.3) | 0.125 |
|  | 118 (18.9) | 40 (19.4) | 0.865 |
|  | 31 (5.0) | 15 (7.3) | 0.206 |
|  | 38 (6.1) | 13 (6.3) | 0.905 |
|  | 22 (3.5) | 4 (1.9) | 0.259 |
| In hospital mortality | 143 (22.8) | 83 (40.3) | **0.0001** |
| CFR 30 days | 158 (25.3%) | 83 (40.3%) | **0.0001** |
| CFR 90 days | 210 (33.6%) | 91 (44.2%) | **0.01** |
| CFR one year | 258 (41.3%) | 95 (46.1%) | 0.31 |
| CFR two year | 285 (45.6%) | 105 (51.0%) | 0.24 |

*Supplementary table four: Descriptive characteristics by ischaemic vs intracerebral haemorrhage strokes, independent t tests, chi2 test.*

|  | **N** | **Received neuroimaging**  **857 (87%)** | **Did not receive neuroimaging**  **129 (13%)** | **P value** |
| --- | --- | --- | --- | --- |
| First in lifetime stroke | 128 | 111 (86.7) | 17 (13.3) | 0.99 |
| **Male** | **495 (50.2)** | **442 (51.6)** | **53 (10.7)** | **0.03** |
| Age mean (SD) | 58.9 (14.0) | 58.6 (14.0) | 60.9 (13.5) | 0.08 |
| Resident of Freetown | 822 (84) | 719 (87.5) | 103 (12.5) | 0.75 |
| Hypertension | 831 (84.3) | 720 (86.6) | 111 (13.4) | 0.70 |
| Diabetes | 212 (21.5) | 177 (83.5) | 35 (16.5) | 0.20 |
| **Atrial Fibrillation** | **38 (3.9)** | **33 (86.8)** | **5 (13.2)** | **0.04** |
| Higher education level (finished high school)  Primary breadwinner  Prescribed Regular medicine before stroke (any) | 367 | 324 (88.3) | 43 (11.7) | 0.57 |
|  | 424 | 378 (89.2) | 46 (10.8) | 0.09 |
|  | 523 | 444 (84.9) | 79 (15.1) | 0.07 |
| Pre-stroke Barthel Index Mean and (SD) | 96.7 (12.4) | 96.7 (12.6) | 96.7 (11.1) | 0.99 |
| **Mean National Institute of Health Stroke Severity Scale (SD)** | 16.6 (9.2) | **15.2 (8.5)** | **26.0 (8.1)** | **0.0001** |
| **In hospital mortality** | **355** | **243 (68.5)** | **112 (31.5)** | **0.0001** |

*Supplementary table five: Descriptive characteristics by patients who received neuroimaging vs those who did not, independent t tests, chi2 test.*

Out of the 129 patients who did not receive a CT scan or MRI, 109/129 died in hospital, the reasons remaining patients did not receive CT scanning were, either the CT scan machines or ambulances were non-functional at that time (n=14), or they discharged against medical advice before imaging (n=4) or declined to be scanned (n=2).

| **Independent Variable** | **Hazard Ratio** | **95% CI** |
| --- | --- | --- |
| Age (each additional year)* | 1.00 | 1.00-1.01 |
| NIHSS (each additional point)* | 1.08 | 1.06-1.09 |
| **Male sex** | **1.25** | **1.03-1.52** |
| **Previous stroke** | **1.37** | **1.08-1.76** |
| **Hypertension** | **0.71** | **0.56-0.90** |
| Diabetes | 1.06 | 0.85-1.30 |
| **Atrial fibrillation** | **1.55** | **1.04-2.29** |
| Higher education level | 0.95 | 0.84-1.07 |
| Primary breadwinner | 0.89 | 0.73-1.09 |
| ¥Intracerebral haemorrhage | 1.22 | 0.97-1.55 |
| ¥**Subarachnoid haemorrhage** | **2.50** | **1.52-4.12** |
| ¥**Undetermined stroke type** | **3.61** | **2.79-4.67** |

*Supplementary table six: Sensitivity analysis of Cox Proportional Hazards model for all strokes, excluding complications. *Control variables.* ¥*Stroke type compared to ischaemic stroke n=986.*

*Supplementary Figure two: Kaplan-Meier estimates for stroke survival, with censoring marks. 1a: Kaplan-Meier survival estimate for all strokes (n=986). 1b Kaplan-Meier survival estimate by sex (n=986). 1c Kaplan-Meier survival estimate by stroke type (n=986). 1d Kaplan-Meier survival estimate by age <55 years vs ≥55 years (n=986).*
